# *Pentatrichomonas hominis* induces extracellular traps formation of macrophages via the TLR2/NADPH/PAD4 pathway

**DOI:** 10.1186/s13071-025-06840-w

**Published:** 2025-07-01

**Authors:** Qi-an Zhai, Xi-chen Zhang, Hong-bo Zhang, Jian-hua Li, Peng-tao Gong, Xiao-cen Wang, Xin Li, Xu Zhang, Nan Zhang

**Affiliations:** State Key Laboratory for Diagnosis and Treatment of Severe Zoonotic Infectious Diseases, Key Laboratory for Zoonosis Research of the Ministry of Education, Institute of Zoonosis, and College of Veterinary Medicine, Jilin University, Changchun, 130062 People’s Republic of China

**Keywords:** *Pentatrichomonashominis*, Macrophage extracellular traps, TLR2, NADPH oxidase, PAD4

## Abstract

**Background:**

*Pentatrichomonas hominis* (*P. hominis*) is a newly identified pathogenic zoonotic protozoan belonging to the Trichomonadidae family. *P. hominis* mainly parasitizes the cecum and colon of humans and other mammals, and it can cause diarrhea. Recently, macrophage extracellular traps (METs) have been shown to play an important role in resistance to parasitic infections. However, it remains unclear whether the release of METs by macrophages contributes to *P. hominis* resistance, and the underlying mechanism of this association has yet to be elucidated.

**Methods:**

Scanning electron microscopy (SEM) and immunofluorescence staining were used to determine whether *P. hominis* induced the formation of METs in mouse peritoneal macrophages to capture and immobilize the parasite as well as the components of METs, including the DNA backbone, myeloperoxidase (MPO), and histone H3. Reactive oxygen species (ROS) and signaling pathway inhibitor assays revealed that the mechanism of *P. hominis*-induced MET formation was dependent on nicotinamide adenine dinucleotide phosphate hydrogen (NADPH) oxidase activation, store-operated calcium entry (SOCE),, and peptidyl arginine deiminase 4 (PAD4) activation. The toll-like receptors 2 (TLR2), extracellular regulated protein kinase 1/2 (ERK1/2) and p38 MAPK signaling pathway were also involved in this process. Trypan blue staining revealed a gradual decrease in the survival rate of *P. hominis* as the coculture time increased. Trypan blue staining also revealed an increase in the proportion of macrophages.

**Results:**

The results of this study indicate that *P. hominis* can induce the release of METs via the TLR2/NADPH/PAD4 pathways and that METs have a trapping and killing effect on *P. hominis*.

**Conclusions:**

This was the first study to reveal that PAD4 and TLR2 were found to be involved in the development of parasite-induced METs, thus providing guidance for further research on the mechanisms of host innate immunity against parasitic infection.

**Graphical Abstract:**

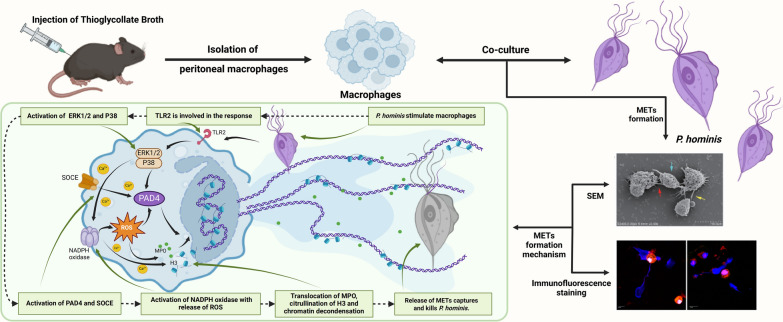

## Background

*Pentatrichomonas hominis* (*P. hominis*) is a zoonotic protozoan belonging to the Trichomonadidae family that is predominantly a trophozoite throughout its life cycle. *P. hominis* is transmitted via the fecal‒oral route and is primarily parasitic in the cecum and colon of humans and other mammals [1]. Most studies on *P. hominis* have been limited to epidemiological investigations, and the results have shown that *P. hominis* can infect domestic, laboratory, companion and wild animals, with a high prevalence in pigs, cattle, goats and dogs [2–6]. In the past, *P. hominis* was considered as a nonpathogenic or conditionally pathogenic parasite, but further research has revealed that *P. hominis* can cause abdominal pain and diarrhea in animals. Moreover, the ability of *P. hominis* to adhere to intestinal epithelial cells and damage the intestinal barrier has led to the identification of *P. hominis* as a pathogenic parasite [2, 7–11]. Additionally, the study has shown that *P. hominis* infection is strongly correlated with the development of colon cancer, and the prevalence of *P. hominis* infection in patients with colon cancer is as high as 41.54% [12]. Although *P. hominis* has attracted increasing attention, few studies have examined the innate immune system’s defense against *P. hominis* infection.

The key components of the innate immune response include phagocytosis of pathogens, induction of inflammation, production of antimicrobial molecules, and formation of extracellular trap networks. Among them, extracellular traps (ETs) have attracted considerable attention in recent years, and many studies have shown that ETs play an important role in host defense against pathogen invasion [13–19]. Moreover, immune cells such as neutrophils, macrophages, mast cells and eosinophils produce ETs [20–28]. In 2010, mature differentiated macrophages were reported to produce ETs called macrophage extracellular traps (METs) [29]. Previous studies on METs have focused on noninfectious diseases and bacterial and fungal infections [30–39], and a growing number of parasites have been shown to induce the release of METs from macrophages, which can trap and kill parasites [34, 40]. The induction of METs by luminal parasitic protozoa such as *Giardia lamblia* and *Trichomonas vaginalis* has been previously reported, *Giardia lamblia* and *Trichomonas vaginalis* can induce METs by activating MAPK signaling pathways dependent on nicotinamide adenine dinucleotide phosphate hydrogen (NADPH) and store-operated calcium entry (SOCE) [35, 36]. However, the underlying mechanisms have not been investigated. Recent studies have shown that pattern recognition receptors (PRRs) tend to be involved when ETs are induced by other stimuli and that Toll-like receptors (TLRs) are the PRRs most often involved in ETs [41–43]. Only a few parasite-related studies have shown that TLR2, TLR4, and TLR9 are involved in parasite-induced neutrophil extracellular traps [44–46], whereas the role of PRRs in parasite-induced METs has not been reported. A growing body of research suggests that peptidyl arginine deaminases (PADs) play crucial roles in the release of METs [47–49] and that peptidyl arginine deiminase 4 (PAD4) citrullinates histone proteins. This process of citrullination leads to structural and charge changes in histones, thereby promoting chromatin deconcentration and the release of DNA from macrophages into the extracellular space [49, 50]. However, this pathway has not been reported for parasite-induced METs. Therefore, the role of PAD4 in the development of parasite-induced METs needs to be further explored.

The aim of this study was to investigate whether *P. hominis* can induce the formation of METs in mouse peritoneal macrophages and observe the components of METs as well as the mechanisms of the signaling pathways, such as the TLR2, NADPH, and PAD4 pathways. Furthermore, the purpose of this study is to increase knowledge regarding the mechanisms of host innate immunity in resisting parasitic infections.

## Methods

### Ethics statement

Female C57BL/6 mice were purchased from the Changsheng Experimental Animal Center (Changchun, China) and raised in the National Experimental Teaching Demonstration Center of Jilin University (Changchun, China). All experimental procedures connected with *P. hominis* were strictly operated in a biological safety cabinet and complied with pathogenic microorganisms laboratory biosafety regulations of China. All animal experimental procedures were performed in strict accordance with the Regulations for the Administration of Affairs Concerning Experimental Animals approved through the State Council of People’s Republic of China and with approval of the Animal Welfare and Research Ethics Committee at Jilin University (SY202201103).

### *P. hominis* trophozoites culture and collection

*P. hominis* strains (ATCC30000) were frozen in the Laboratory of Parasitology, School of Zoology, Jilin University. *P. hominis* was cultured aseptically to logarithmic growth phase in TYI-S-33 medium at 37 °C. Collected trophozoites were washed by Roswell Park Memorial Institute (RPMI) 1640 medium (Biological Industries, 01-100-1A, Israel) and resuspended in RPMI 1640 until further experimental use.

### Isolation of mouse peritoneal macrophages

The mice were injected with 3 ml of thioglycollate broth (Thermo Fisher, CM0391B, USA) at 3 days before the experiment. For macrophage collection, the mice were euthanized via cervical dislocation and soaked in 75% ethanol for 15 min. The peritoneal cavities were gently flushed twice with 15 ml of 1× PBS (Sangon Biotech, E607008, China), and the cells were collected by centrifugation at 1,000*g* for 10 min. Macrophages were washed twice with 20 ml of 1× PBS, resuspended in RPMI 1640, and incubated overnight at 37 °C with 5% CO_2_. The cells were washed twice with PBS to remove the nonadherent cells.

### Flow cytometry

Macrophages were seeded into six-well plates. Following attachment, the cells were washed three times with PBS to remove any nonadherent cells or debris. The adherent cells were subsequently digested with 1% trypsin at 37 °C for 5 min. The cell suspension was then centrifuged at 300*g* for 8 min. A total of 1 × 10⁶ cells were transferred to a 1.5-ml centrifuge tube. For surface staining, 0.5 μg/test of PE-conjugated rat anti-mouse F4/80 (BD Pharmingen, 565410, USA) and PerCP-Cy5.5-conjugated rat anti-CD11b (BD Pharmingen, 561114, USA) were added to the cell suspension and incubated in the dark at 4 °C for 30 min. After staining, the cells were washed with stain buffer to remove unbound antibodies and resuspended in 200 μl of PBS for flow cytometric analysis.

### Scanning electron microscopy (SEM)

Mouse macrophages (2 × 10^5^ cells/ml) were precultured on 1 mg/ml poly-l lysine (Merck, P8920, USA)-coated glass in 24-well culture plates overnight and then incubated with *P. hominis* (4 × 10^5^ trophozoites/ml) at 37 °C for 120 min. Nonadherent cells and trophozoites were washed with RPMI 1640. The cells were fixed with 4.0% glutaraldehyde (Merck, 354400, USA) for 24 h, gently washed with PBS, and postfixed with 1.0% osmium tetroxide (Merck, 251755, USA). The fixed samples were first dehydrated in increasing ethanol concentrations (30%, 50%, 70%, 80%, 90%, and 100%), vacuum dried, and sputtered with a conductive coating. The samples were then observed via SEM (Hitachi, Japan).

### Immunofluorescence staining analysis

Mouse macrophages (2 × 10^5^ cells/ml) were cultured in polylysine-coated cell culture dishes. *P. hominis* (4 × 10^5^ trophozoites/ml) were stained with 25 μM cell tracker green CMFDA (MKBio, MX4107, China), incubated with cells after cell attachment, fixed in 4% paraformaldehyde for 15 min, permeabilized with 0.25% Triton X-100 for 20 min, washed three times with sterilized PBS, and blocked with 3% bovine serum albumin (BSA; Merck, V900933, USA). The samples were incubated with myeloperoxidase (MPO; 1:200, MedChemExpress, HY-P990181, USA) or histone H3 (1:200, Abcam, ab1791, UK) incubated at 4 °C overnight, washed three times with sterilized PBS, and further incubated with Cy3-conjugated goat anti-rabbit immunoglobulin (Ig)G (1:100, Proteintech, SA00009-2, USA). Hoechst 33342 (Beyotime, P0133, China) was used to detect DNA released by the macrophages. The samples were then observed under a fluorescence confocal microscope (Olympus Corporation, Japan).

### Quantitative real-time PCR

Mouse macrophages (7.5 × 10^5^ cells/ml) were cultured in six-well plates and stimulated with *P. hominis* or the positive stimulant zymosan (Merck, Z4250, USA). Total RNA was extracted and reverse transcribed into cDNA after the cells were collected at different time points (30, 60, 90, and 120 min). The cDNA was amplified via SYBR Green quantitative polymerase chain reaction (qPCR) Master Mix (Thermo Fisher, A66732, USA) to detect the mRNA of *Tlr2* (primers: *Tlr2*-F 5′-CCCACTTCAGGCTCTTTGAC-3′ and *Tlr2*-R 5′-GCCACTCCAGGTAGGTCTTG-3′). The amplification procedure was performed as follows: initial denaturation at 95 °C for 5 min; 40 cycles of denaturation at 94 °C for 30 s, annealing at 60 °C for 30 s, and extension at 72 °C for 30 s, with a final extension at 72 °C for 10 min. The internal control gene used was β-actin (*Actb*).

### Quantitative analysis of METs

Mouse macrophages (7.5 × 10^5^ cells/ml) were precultured overnight in 96-well plates. The cells were then incubated with *P. hominis* trophozoites at different ratios (macrophages: *P. hominis*, 50:1, 20:1, 10:1, 5:1, 2:1, 1:1, 1:2, and 1:4) and for different time points (30, 60, 90, 120, 150, and 180 min). The cells incubated with 1 mg/ml zymosan (different time grouping: 30, 60, 90, 120, 150, and 180 min; different ratios grouping: 120 min) or culture medium were used as positive and negative controls, respectively. In parallel experiments, macrophages were pretreated with 20 μM of NADPH oxidase inhibitor (diphenylene iodonium [DPI], Merck, D2926, USA), 100 μM of MPO inhibitor (4-aminobenzoic acid hydrazide [4-ABAH], Selleck, S9874, China), 50 μM of extracellular regulated protein kinase 1/2 (ERK1/2) inhibitor (U0126, Selleck, S1102, China), 10 μM of p38 MAPK inhibitor (SB202190, Selleck, S1077, China), 100 μM of TLR2 inhibitor (TLR2-IN-C29, Selleck, S6597, China), 100 μM of SOCE inhibitor (2-aminoethyl diphenylborinate [2-APB], Selleck, S6657, China), 10 μM of PAD4 inhibitor (GSK484 hydrochloride, MedChemExpress, HY-100514, USA), and 1 μM of cytochalasin D (Cyt D, APExBio, 22144-77-0, USA) at 37 °C and 5% CO_2_ for 30 min. DNase I in 90 U/well (Thermo Scientific, EN0523, USA) was used to digest the MET structure for 15 min before the end of coincubation. The double-stranded DNA (dsDNA) content in the cell culture supernatant was measured via the Quant-iT picogreen dsDNA reagent and kit (Invitrogen, Q33130, USA). The samples were analyzed via a fluorescence microplate reader (BioTek, USA) at an excitation wavelength of 485 nm and an emission wavelength of 530 nm.

### Phagocytic test

Fluorescein isothiocyanate (FITC)-labeled zymosan particles were prepared. Zymosan was solubilized in 0.1 M sodium carbonate buffer, disrupted by sonication for 5 min, and then centrifuged at 12,000*g* for 10 min. The supernatant was discarded, and the precipitate was resuspended in 0.1 M sodium carbonate buffer (pH 9.5) and vortexed for 2 min. Fluorescent labeling was performed by incubating zymosan (20 mg/ml) with FITC (5 mg/ml) in carbonate buffer at 37 °C for 30 min. After labeling, the zymosan was washed twice with carbonate buffer and twice with incomplete RPMI. After these treatments, zymosan was considered zymosan-FITC.

We examined the phagocytic activity of macrophages using FITC-conjugated zymosan. Peritoneal macrophages were plated at a cell density of 1 × 10^5^ cells/well in phenol red-free RPMI 1640 and incubated for 24 h at 37 °C in 5% CO_2_. The cells were then incubated for 1 h with Cyt D; 20 μl of FITC-zymosan was added and incubated for 1 h. After washing with medium three times, 100 μl of triton X-100 (10%) was added and the cells were vigorously shaken to lyse them. After lysis, the mixture was centrifuged at 12,000*g* for 10 min at 4 °C. The supernatant from each well was collected, and the fluorescence was measured using a fluorescence microplate reader at an excitation wavelength of 485 nm and an emission wavelength of 530 nm.

### Detection of reactive oxygen species (ROS)

After the macrophages were pretreated with or without NADPH oxidase inhibitor (DPI), TLR2 inhibitor (C29), and p38 MAPK inhibitor (SB202190), they were incubated with *P. hominis* (macrophage: *P. hominis*, 1:2) and zymosan (1 mg/ml) at 37 °C for 120 min. The cells stimulated with zymosan (1 mg/ml) and RPMI 1640 were used as positive and negative controls, respectively. The samples were then incubated with 2',7'-dichloro-fluorescein diacetate (DCFH-DA; Merck, D6883, USA), and the fluorescence intensity was detected via a fluorescence microplate reader at 488 nm excitation and 525 nm emission wavelengths.

### Lactate dehydrogenase (LDH) release assay

Mouse macrophages were inoculated in 96-well cell culture plates at a ratio of 2.0 × 10^5^ cells/ml and stimulated with *P. hominis* at a ratio of 1:2 (macrophages: *P. hominis*) for 30, 60, 90, and 120 min. Cells incubated with lysis buffer were used as a positive control, and the culture medium was used as a negative control. Cell supernatants were collected and LDH levels were measured using the LDH cytotoxicity assay kit (Beyotime, C0019S, China) according to the manufacturer’s instructions.

### Determination of ***P. hominis*** viability

Mouse macrophages (7.5 × 10^5^ cells/ml) were incubated with *P. hominis* trophozoites at different ratios (macrophages: *P. hominis*, 1:1, 2:1 and 4:1) in 24-well cell culture plates for different time periods (60, 120, and 180 min). After 45, 105, and 165 min of coincubation, DNase I was used to digest the MET structure for 15 min. The culture medium was centrifuged at 1,000*g* for 10 min, resuspended in PBS containing 0.4% trypan blue (Solarbio, C0040, China), and stained for 3 min, after which, trophozoite survival was observed under a microscope. The survival rate = (number of negative trophozoites stained with trypan blue/total number of trophozoites) × 100%.

### Statistical analysis

Data were analyzed using one-way analysis of variance (ANOVA) or Dunnett’s test, and the experiment was repeated three times. All data analyses were performed using the GraphPad Prism 9 software. Data are shown as mean ± standard deviation (SD). Differences between groups were considered significant at *P* < 0.05 (*), *P* < 0.01 (**), *P* < 0.001 (***), and *P* < 0.0001 (****).

## Results

### *P. hominis* trophozoites induce the formation of METs in mouse macrophages

**Fig. 1:**
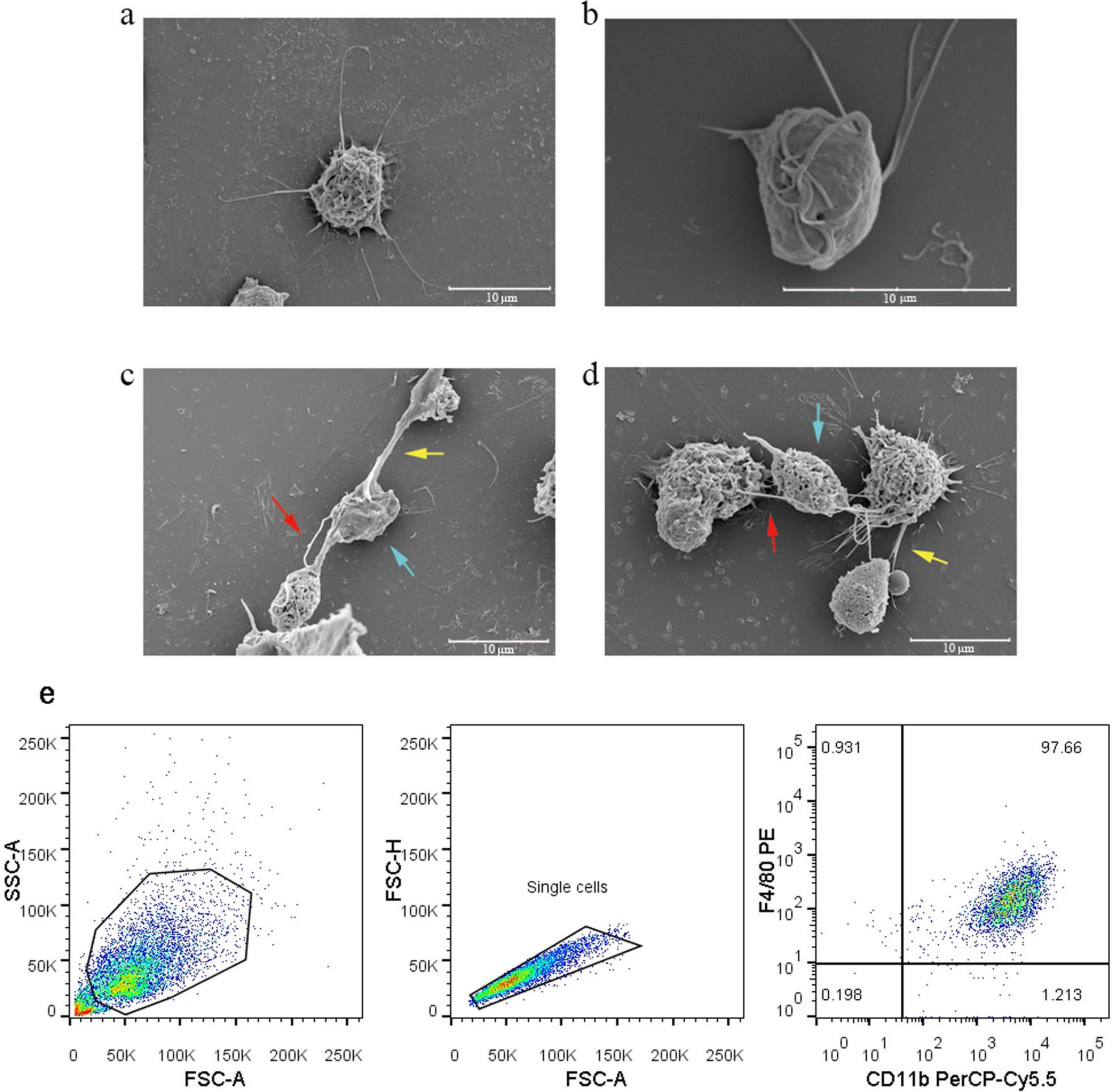
Scanning electron micrography image of METs in mouse peritoneal macrophages triggered by *P. hominis* and flow cytometry for assessing macrophage purity. The macrophages (2 × 10^5^ cells/ml) were incubated with 4 × 10^5^ *P. hominis* trophozoites or RPMI 1640 (negative control) at 37 °C for 120 min. **a**, Mouse macrophages. **b**, *P. hominis* trophozoites. **c**,**d**, Extracellular net-like structures released by macrophages to capture *P. hominis*. Yellow arrows indicate net-like structures, blue arrows indicate live trophozoites, and red arrows indicate flagella. Scale bar for **a**–**d**, 10 μm. **e**, Macrophages were stained with F4/80 and CD11b antibodies, and flow cytometry was used to determine the purity of macrophages, which was found to be 97.66%

Mouse macrophages were incubated with *P. hominis* trophozoites for 120 min (ratio of 1:2) and MET formation was determined via SEM and fluorescence confocal microscopy. Scanning electron microscopic analysis revealed that exposure to *P. hominis* trophozoites triggered the formation of filamentary structures by mouse macrophages (Fig. 1a–d). These extracellular reticular structures tightly adhered to and trapped the surface of the *P. hominis* trophozoites. To identify the cell type and purity of those producing the reticular structures, we used F4/80 and CD11b as markers for macrophage purity analysis. Flow cytometry showed a macrophage purity of 97.66% under the same conditions, indicating that most cells releasing reticular structures after incubation with the parasite were macrophages (Fig. 1e).

**Fig. 2:**
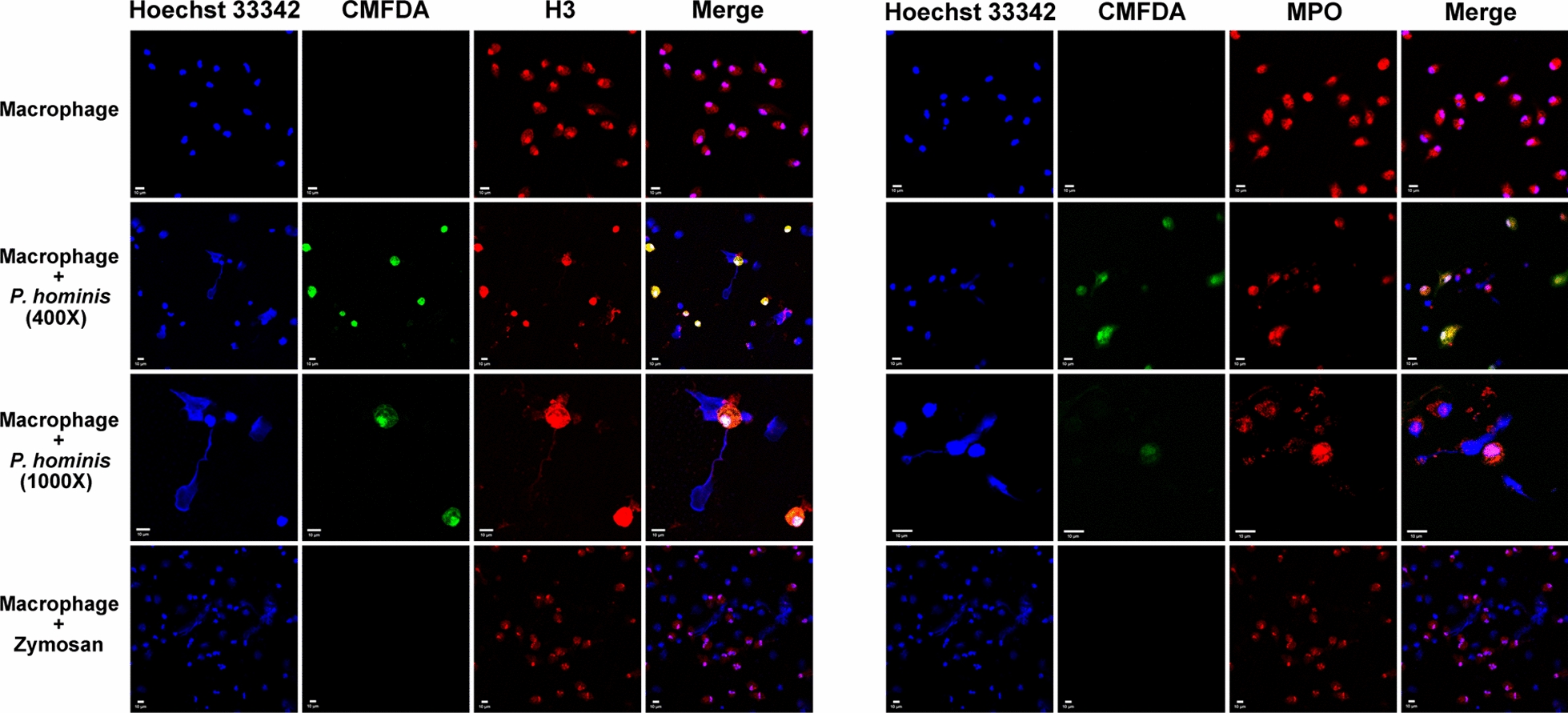
Visualization of the DNA skeleton decorated with H3 and MPO. The macrophages (2 × 10^5^ cells/ml) were precultured in polylysine-coated cell culture dishes and stimulated with *P. hominis* (4 × 10^5^ trophozoites/ml) for 120 min (37 °C, 5% CO_2_). The DNA backbone of METs was stained with Hoechst 33,342 (blue). H3 and MPO were stained with a histone H3 or MPO antibody followed by Cy3 goat anti-rabbit IgG (red). *P. hominis* were stained with cell-tracker green CMFDA (green). Scale bar, 10 μm

To confirm whether these reticular structures were ETs released by macrophages, immunofluorescence staining was performed, as the ETs commonly contain a DNA backbone colocalized with antimicrobial and granule proteins, including histone H3 and MPO [51–53]. Fluorescence confocal microscopic analysis revealed that DNA can be observed by Hoechst 33342 staining to form a filamentous mesh that is released from the nucleus and wrapped around *P. hominis* trophozoites stained with CMFDA and immunofluorescence staining with specific H3 and MPO antibodies detected the colocalization of DNA with H3 and MPO (Fig. 2). Thus, *P. hominis* trophozoites can trigger MET formation.

### *P. hominis*-induced MET formation is in a time-dependent manner

**Fig. 3:**
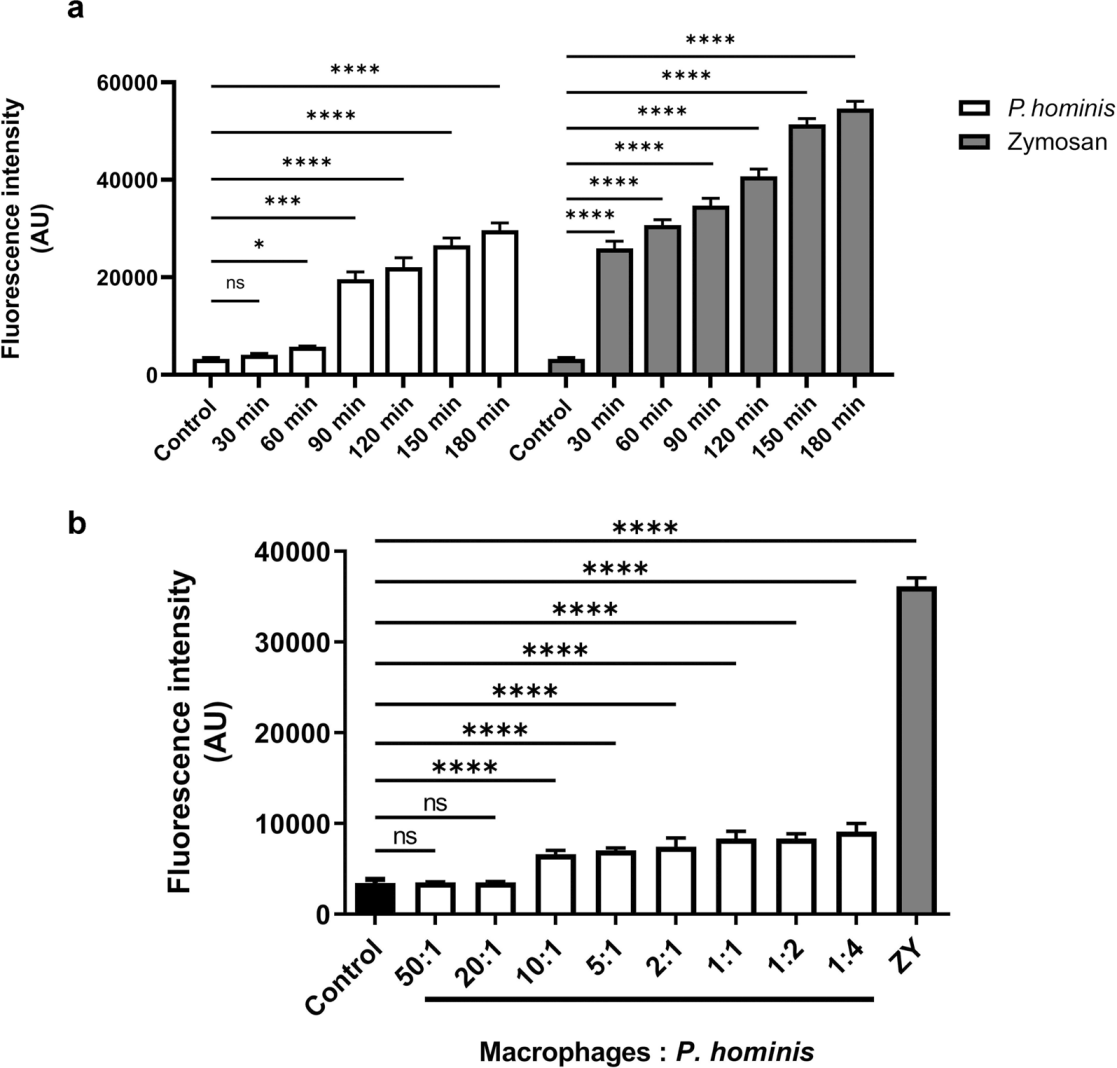
MET formation triggered by *P. hominis* was in a time- and dose-independent manner. **a**, Macrophages (7.5 × 10^5^ cells/ml) were stimulated with *P. hominis* (1.5 × 10^6^ cells/ml) and zymosan (1 mg/ml) for 30, 60, 90, 120, 150, and 180 min. **b**, Macrophages (7.5 × 10^5^ cells/ml) were stimulated with *P. hominis* at ratios of 50:1, 20:1, 10:1 5:1, 2:1, 1:1, 1:2, and 1:4 (macrophages:*P. hominis*) and zymosan for 120 min. After coincubation, the dsDNA produced by METs in the supernatants was stained with PicoGreen and detected (excitation wavelength of 485 nm; detection wavelength of 530 nm). The unstimulated macrophages were used as a negative control. Bars represent the mean ± SD for the three experiments. ZY, Zymosan; ns, not significant. *P < 0.05, ***P < 0.001, ****P < 0.0001

*P. hominis*-induced METs were quantified via a PicoGreen dsDNA kit. Compared with that of the negative control, the quantity of METs stimulated by *P. hominis* significantly increased after 60 min of coincubation; however, the production level was lower than that of the positive control. With prolonged stimulation, the fluorescence intensity increased significantly (Fig. 3a). When the ratio of macrophages to *P. hominis* was 10:1, the fluorescence intensity significantly increased; however, with increasing parasite number, no significant increase in fluorescence intensity was observed (Fig. 3b). These results indicate that *P. hominis*-induced MET formation was in a time-dependent manner rather than a dose-dependent manner.

### *P. hominis* have no impact on LDH levels during METosis

**Fig. 4:**
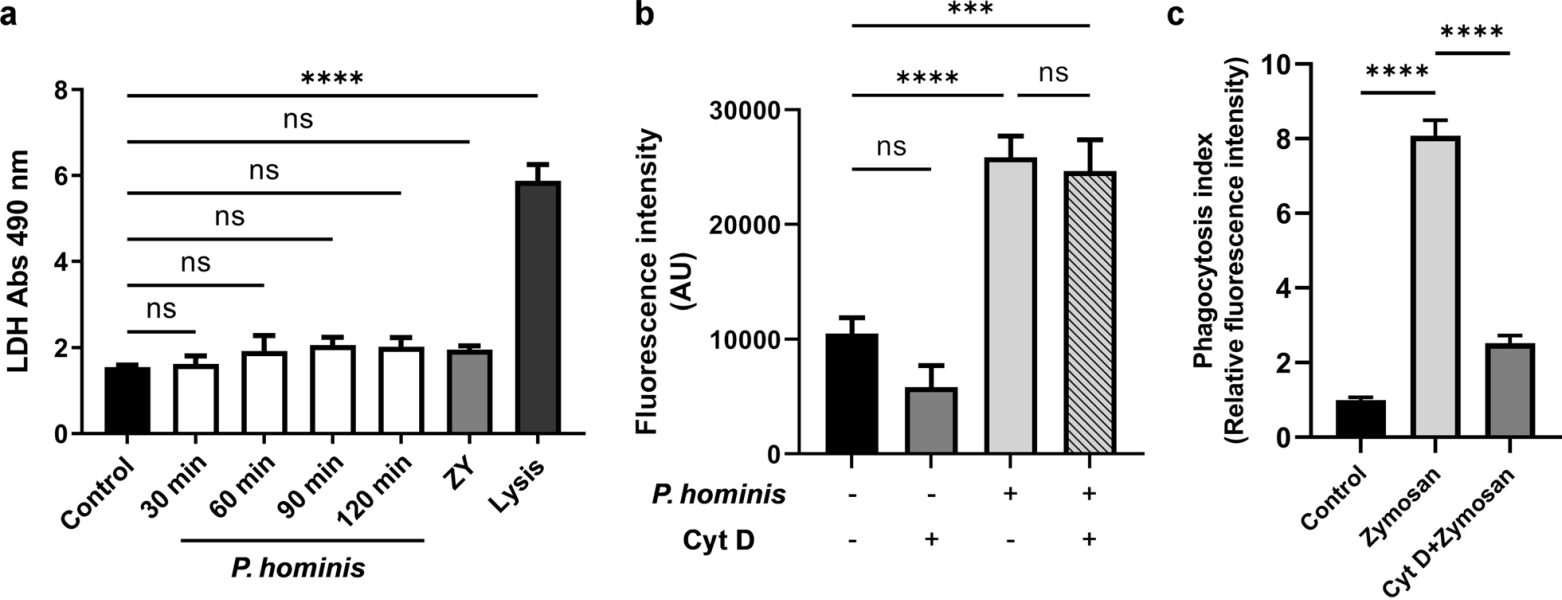
*P. hominis*-induced METs had no effect on LDH and phagocytosis has no effect on METs. **a**, Macrophages (2.0 × 10^5^ cells/ml) were stimulated with *P. hominis* (2:1 ratio) for 30, 60, 90, and 120 min. Cellular LDH activity in the cell supernatant was measured via an LDH assay kit. Lysis was used as a positive control and unstimulated macrophages were used as the negative control. **b**, Cyt D pretreatment of macrophages (7.5 × 10^5^ cells/ml) for 30 min, after which they were stimulated with *P. hominis* (1:2 ratio) for 120 min. The dsDNA produced by METs in the supernatants was stained with PicoGreen and detected. Macrophages stimulated with zymosan (1 mg/ml) were used as a positive control, and unstimulated macrophages were used as a negative control. **c** Examined the phagocytic activity of macrophages using FITC-zymosan, detected with excitation wavelength of 485 nm; detection wavelength of 530 nm. ZY: Zymosan. ns, not significant, ***P < 0.001, ****P < 0.0001

In recent years, emerging evidence has suggested that ETosis is a novel form of cell death that is distinct from necrosis. ETosis does not trigger the release of LDH, which is an enzyme released into the surrounding environment during cell necrosis. To determine whether MET formation induced by *P. hominis* trophozoites is different from necrosis, we examined LDH activity during *P. hominis*- and zymosan-induced MET formation. The results revealed that there was no significant change in LDH levels in macrophages exposed to *P. hominis* trophozoites for various time periods compared with control (Fig. 4a). Thus, the process of MET formation is similar to that of NETosis but different from that of necrosis.

### Cell phagocytosis has no effect on *P. hominis*-induced METs

To exclude the effect of macrophage phagocytosis in this assay, cytochalasin D was added 30 min before coincubation. The cells were stimulated with *P. hominis* or incubated with zymosan for 120 min, after which the supernatant was collected and the release of extracellular DNA was detected via a fluorescence microplate reader. The results revealed that cytochalasin D treatment did not affect the ability of *P. hominis* to induce the extracellular release of DNA from macrophages, and the treated macrophages were still able to release a certain amount of DNA compared with the controls (Fig. 4b). Through the macrophage phagocytosis inhibition assay, we demonstrated that Cyt D was active (Fig. 4c).

### *P. hominis* can cause NADPH oxidase activation and ROS production during MET formation

**Fig. 5:**
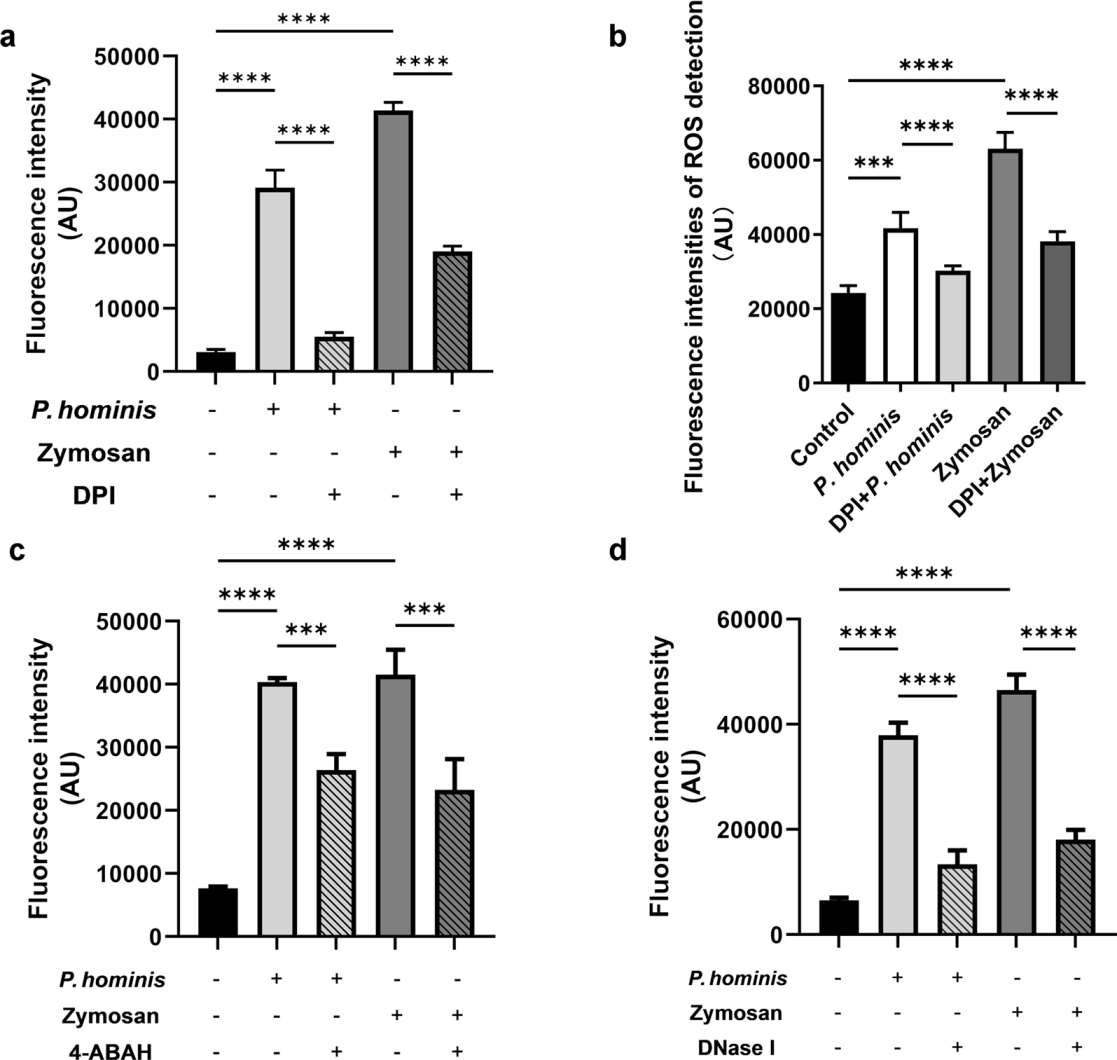
NADPH activation and ROS production were involved in the formation of *P. hominis*-induced METs. **a**, Macrophages (7.5 × 10^5^ cells/ml) were pretreated with NADPH oxidase inhibitor (DPI) prior to *P. hominis* trophozoite stimulation for 30 min. **b**, ROS production was detected via DCFH-DA. Macrophages were pretreated or untreated with DPI. The macrophages stimulated with zymosan (1 mg/ml) were used as a positive control, and unstimulated macrophages were used as a negative control. **c**, Macrophages (7.5 × 10^5^ cells/ml) were pretreated with MPO inhibitor (4-ABAH) prior to *P. hominis* trophozoite stimulation for 30 min. **d**, DNase I was added to the coincubated medium 15 min before the end of incubation. The dsDNA produced by METs in supernatants was stained with PicoGreen and detected. Bars represent the mean ± SD for the three experiments, ***P < 0.001, ****P < 0.0001

ET release processes are closely related to the activation of NADPH oxidase stimulated by pathogens, leading to H3 citrullination and ROS production. ROS production can cause MPO degranulation to migrate around the nucleus and promote chromatin deprotonation to generate ETs [53, 54]. Therefore, we used the NADPH oxidase-specific inhibitor DPI to determine whether *P. hominis* trophozoites could induce MET formation. The macrophages were pretreated with DPI and coincubated with *P. hominis* trophozoites. Extracellular DNA was detected via PicoGreen fluorescence staining. As expected, the level of DNA released by macrophages pretreated with DPI was significantly lower than that released by those that did not undergo DPI treatment, demonstrating that the process of MET release stimulated by *P. hominis* trophozoites relies on NADPH oxidase activation (Fig. 5a). Additionally, ROS release assays revealed that stimulation with *P. hominis* trophozoites for 120 min resulted in the release of intracellular ROS from macrophages and pretreatment with DPI inhibited the generation of ROS induced by zymosan and *P. hominis* (Fig. 5b). Consistently, the MPO inhibitor ABAH significantly reduced the amount of DNA released by macrophages coincubated with *P. hominis* trophozoites (Fig. 5c). Moreover, *P. hominis*-induced METs in mouse macrophages were efficiently abolished by DNase I treatment (Fig. 5d), revealing the typical characteristics of METs. These results confirm that *P. hominis* trophozoites can trigger MET formation via NADPH oxidase activation and ROS release-dependent processes.

### TLR2 is involved in the MET formation triggered by *P. hominis* trophozoites

**Fig. 6:**
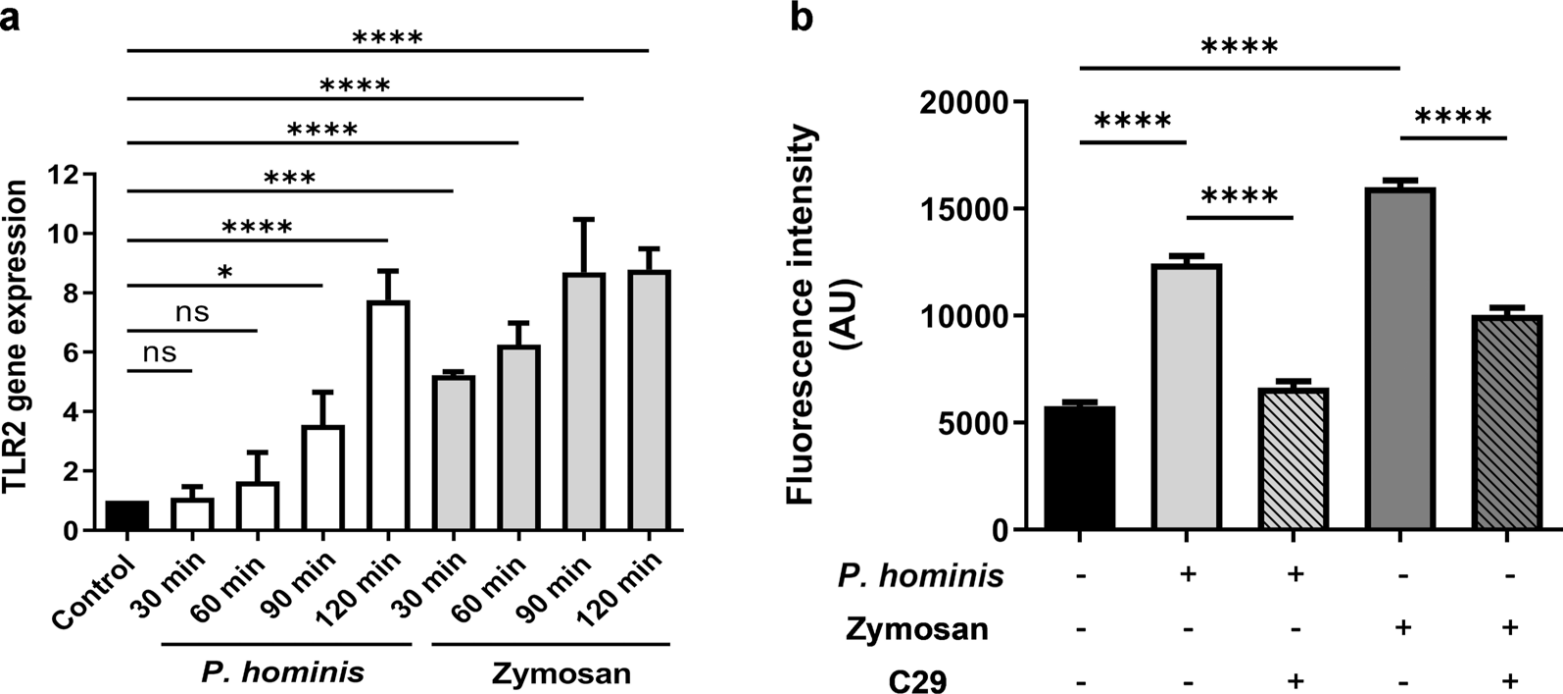
TLR2 was involved in the formation of METs induced by *P. hominis* trophozoites. **a**, To detect the expression of *Tlr2*, macrophages (7.5 × 10^5^ cells/ml) were stimulated with *P. hominis* trophozoites or zymosan for 30, 60, 90, and 120 min. The internal reference gene used was β-actin. **b**, Macrophages were pretreated with the TLR2 inhibitor C29 prior to *P. hominis* trophozoite stimulation. The dsDNA produced by METs in supernatants was stained with PicoGreen and detected. The macrophages stimulated with zymosan were used as positive controls, and the unstimulated macrophages were used as negative controls. Bars represent the mean ± SD for three experiments. ns, not significant. *P < 0.05, ***P < 0.001, ****P < 0.0001

An increasing number of studies have shown that pathogens induce MET via TLR2 [44, 51, 55, 56]. The macrophages were incubated with *P. hominis* trophozoites, and the expression of TLR2 was detected via qPCR. The ETs-positive stimulant zymosan was used as the positive control. The results revealed that the expression of TLR2 in macrophages gradually increased from 30 to 120 min when stimulated with zymosan but significantly increased from 90 to 120 min when stimulated with *P. hominis* trophozoites, thus suggesting that TLR2 is involved in *P. hominis* trophozoite-triggered MET formation (Fig. 6a).

To further investigate the role of TLR2 in the formation of METs induced by *P. hominis* trophozoites, macrophages were pretreated with the TLR2 inhibitor (C29) and then coincubated with *P. hominis*. PicoGreen staining of the extracellular DNA was used to detect MET formation. Compared with the untreated group, the number of METs significantly decreased after pretreatment with the TLR2 inhibitor, thus confirming the role of TLR2 in the formation of METs triggered by *P. hominis* trophozoites (Fig. 6b).

### *P. hominis* triggers MET formation by activating the ERK1/2 and p38 MAPK signaling pathway

**Fig. 7:**
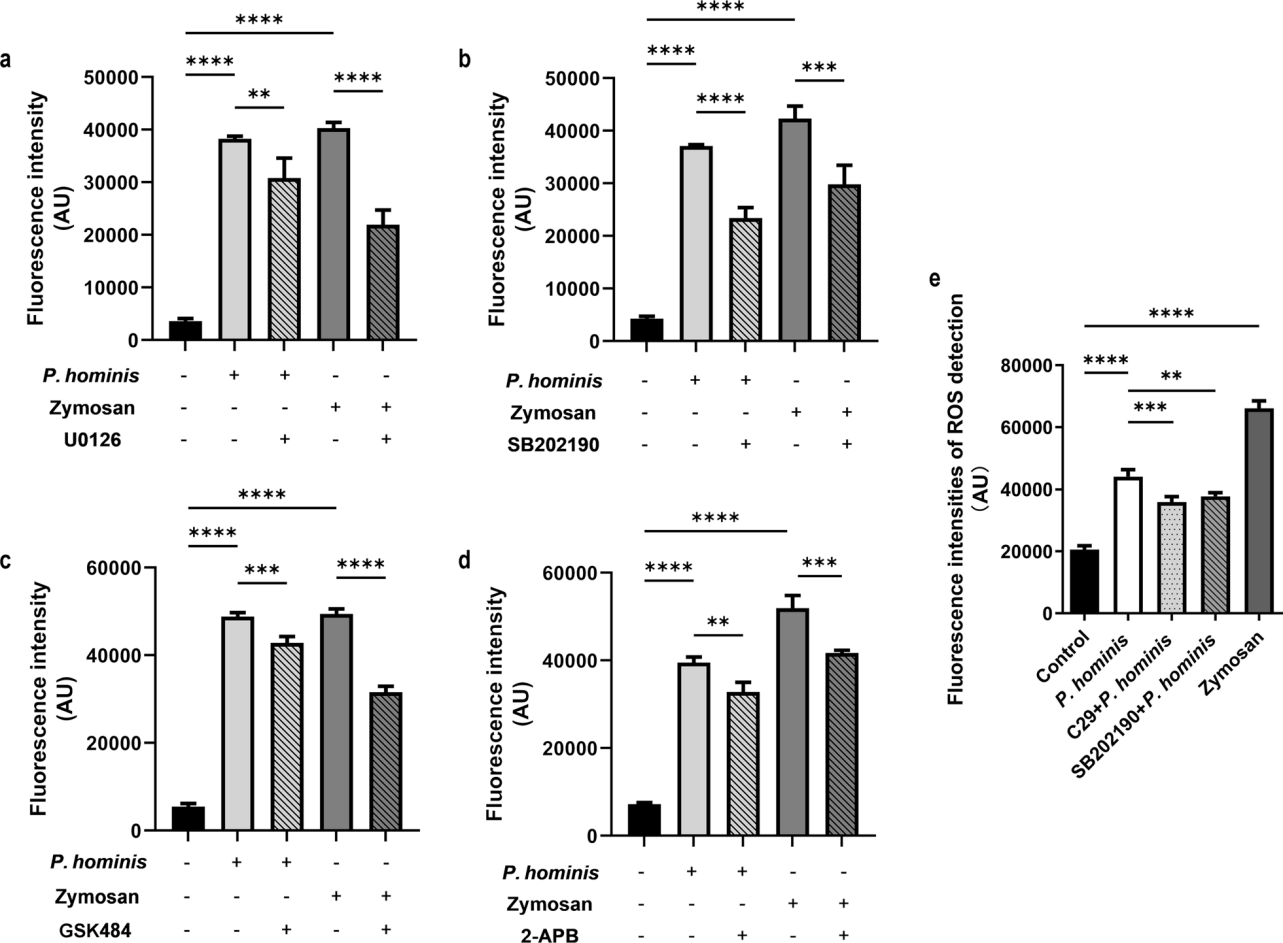
ERK1/2, p38 MAPK signaling pathway, PAD4 and SOCE were involved in *P. hominis*-induced MET formation, TLR2 and p38 MAPK are involved in the induction of ROS. The macrophages were pretreated with: **a**, an ERK1/2 inhibitor (U0126), **b**, a p38 MAPK inhibitor (SB202190), **c**, a PAD4 inhibitor (GSK484), and **d**, a SOCE inhibitor (2-APB) for 30 min and then coincubated with *P. hominis*. The dsDNA produced by METs in supernatants was stained with PicoGreen and detected. **e**, Macrophages were pretreated with C29 and SB202190; ROS production was detected via DCFH-DA. Zymosan-stimulated macrophages were used as a positive control, and unstimulated macrophages were used as a negative control. Bars represent the mean ± SD for three experiments. ns, not significant. *P < 0.05, **P < 0.01, ***P < 0.001, ****P < 0.0001

To understand whether the downstream target pathways of TLR2, the ERK1/2 and p38 MAPK signaling pathway, are involved in the formation of METs triggered by *P. hominis*, macrophages were pretreated with the inhibitor of ERK1/2 (U0126) and the inhibitor of p38 (SB202190), respectively. They were then coincubated with *P. hominis* trophozoites to detect MET production. The results showed that after pretreatment with the inhibitors, the production of extracellular DNA in the macrophages was lower than that in the macrophages that did not receive the treatment (Fig. 7a, b), thus confirming the important role of ERK1/2 and p38 MAPK in triggering MET formation upon *P. hominis* stimulation.

### *P. hominis*-induced MET formation is associated with SOCE and PAD4

Histone citrullination is a key process in MET formation. Arginine is converted to citrulline by PADs, and PAD4 is expressed mainly in immune cells. To determine the role of PAD4 in *P. hominis*-induced MET, macrophages were pretreated with the PAD4 inhibitor (GSK484) and then coincubated with *P. hominis*. The production of METs was observed by detecting the dsDNA content in the supernatant. Compared with that in the control group, the level of extracellular DNA produced by GSK484-pretreated macrophages was significantly lower after *P. hominis* stimulation (Fig. 7c), indicating that the formation of METs triggered by *P. hominis* trophozoites is dependent on PAD4.

SOCE is involved in ET formation [57], and elevated intracellular calcium levels induce Ca^2+^-dependent PAD4 activity, leading to histone citrullination and ROS production [58–62]. Thus, we used a SOCE inhibitor (2-APB) to explore the role of Ca^2+^ in MET formation stimulated by *P. hominis* trophozoites. The level of extracellular DNA was significantly lower in macrophages pretreated with 2-APB than in those that did not receive the treatment (Fig. 7d), suggesting that MET formation triggered by *P. hominis* trophozoites is SOCE-dependent.

### TLR2 and p38 MAPK signal pathway are involved in the induction of ROS release by *P. hominis* in macrophages

ROS release assays revealed that stimulation with *P. hominis* trophozoites for 120 min resulted in the release of intracellular ROS from macrophages, and pretreatment with TLR2 inhibitor (C29) and p38 MAPK inhibitor (SB202190) the generation of ROS induced by *P. hominis* (Fig. 7e). These results suggest that TLR2 and p38 MAPK are involved in the induction of ROS release by *P. hominis* in macrophages.

### METs reduce *P. hominis* viability

**Fig. 8:**
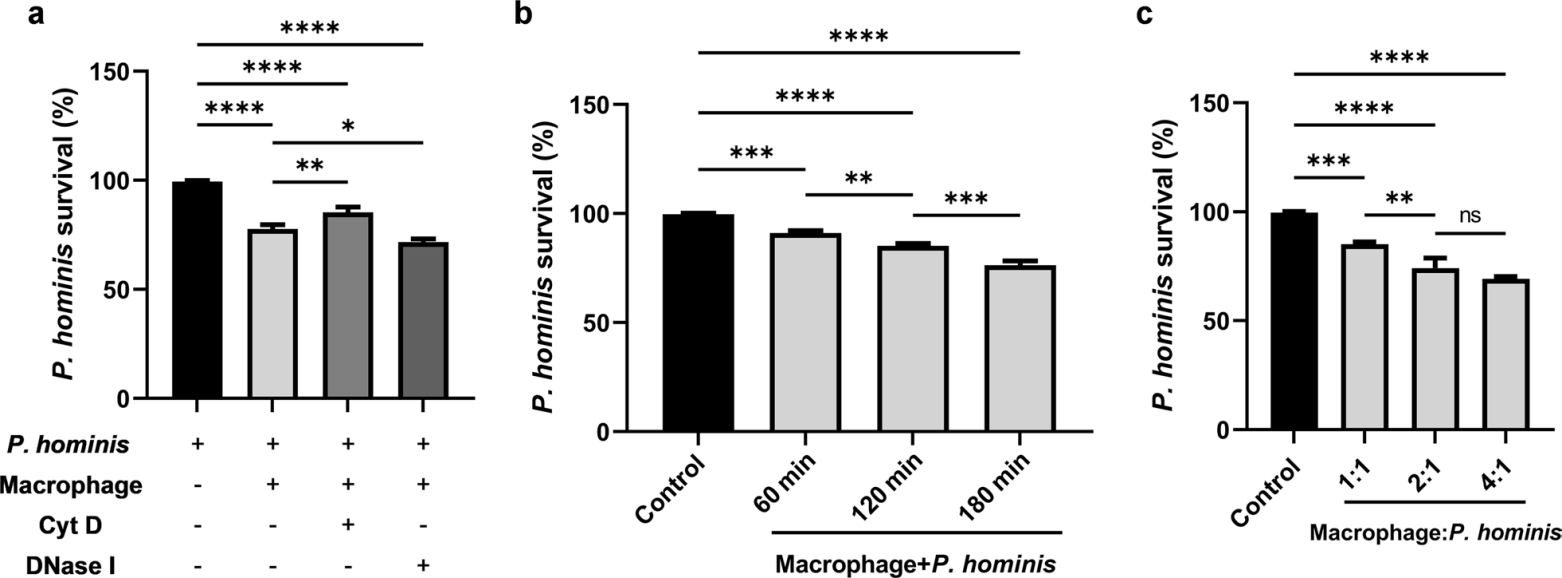
METs reduced *P. hominis* viability. **a**, *P. hominis* (7.5 × 10^5^ cells/ml) was added to the cell-free 1640 medium group, the macrophage (7.5 × 10^5^ cells/ml) group, the Cyt D-pretreated macrophage group, and the DNase I-treated group. **b**, Cyt D-pretreated macrophages (7.5 × 10.^5^ cells/ml) were incubated with *P. hominis* trophozoites at different ratios (macrophages: *P. hominis*, 1:1, 2:1, and 4:1) in 24-well cell culture plates, and **c**, after 45, 105, and 165 min of coincubation, DNase I was used to digest the MET structure for 15 min. The entire medium was collected and centrifuged, and the pellet was resuspended in PBS containing 0.4% trypan blue. Staining was carried out for 3 min, and the trophozoites were analyzed via microscopy to assess viability. The survival rate = (number of negative trophozoites stained with trypan blue/total number of trophozoites) × 100%. The *P. hominis* group cultured in only 1640 medium was used as the control group. Bars represent the mean ± SD for the three experiments. ns, not significant. *P < 0.05, **P < 0.01, ***P < 0.001, ****P < 0.0001

On the basis of microscopic visualization, METs can capture and immobilize *P. hominis*. To further test whether METs had a killing effect on *P. hominis*, after coincubation of *P. hominis* with METs, *P. hominis* was washed and stained with trypan blue to identify its activity. Our results showed that METs significantly reduced the survival rate of *P. hominis*. To rule out the effect of cellular phagocytosis, macrophages were pretreated with Cyt D. Phagocytosis was found to cause the death of some *P. hominis* cells; however, the effect of METs on *P. hominis* was significant when phagocytosis was excluded. To visualize *P. hominis* captured by METs, we used DNase I to digest the DNA mesh released by METs and found that the METs immobilized a portion of *P. hominis* (Fig. 8a).

To further observe the relationship between *P. hominis* cell death due to METs and coincubation time and dose, we evaluated the survival rate of *P. hominis* at different time points and doses. The macrophages were treated with Cyt D and DNase I to prevent phagocytosis and ensure that all *P. hominis* cells were collected. The results revealed that a longer coincubation time was associated with lower *P. hominis* survival, and the lowest survival rate was 76.27% at 180 min (Fig. 8b). A greater proportion of macrophages was associated with a lower *P. hominis* survival rate. Moreover, the lowest survival rate was 69.28% (Fig. 8c). Thus, METs have a killing effect on *P. hominis,* and *P. hominis* survival decreases with time and dose.

## Discussion

The mechanism underlying the effects of ETs was first described in human neutrophils in 2004 [16]. The formation of ETs is a unique series of cellular events in which nuclear contents, including chromatin and cytosolic proteins, are extruded from the cell to form an extracellular network with a DNA backbone containing a variety of active proteins [16, 18]. The formation of ETs is accompanied by immune cell death, and this novel mode of death differs from apoptosis and necroptosis in both cell morphology and mechanism of occurrence [19]. In recent years, this novel effector mechanism has been studied extensively in various innate immune cells and pathogens [20–22]. Macrophages are innate immune cells that are distributed in various mammalian tissues [37] and were first reported to produce METs in 2010 [29]. Previous studies have shown that parasites can induce the production of METs in macrophages, but there are few reports on protozoa parasitizing the lumen of the host. For example, *Trichomonas vaginalis* induces the production of METs in THP-1 cells, and *Giardia lamblia* induces the production of METs in peritoneal macrophages in mice [35, 36]. *P. hominis* is a protozoan parasite of the cecum and colon [8] and has been shown to adhere to intestinal epithelial cells [63]. However, its interaction with macrophages in the intestinal mucosa and whether it induces the production of METs by macrophages have yet to be elucidated. In this study, *P. hominis* was coincubated with mouse peritoneal macrophages, and scanning electron microscopy revealed that *P. hominis* induced the release of net-like structures from mouse macrophages. It is reported that reticular structures have been proven to be released by a variety of immune cells [15, 22, 23], especially neutrophils mentioned in many protozoan studies [17, 18, 26]. To determine whether the observed net-like structures in the study were released by macrophages, we performed flow cytometry using macrophage markers F4/80 and CD11b to assess macrophage purity. The results indicated that macrophages constituted 97.66% of the cell population, which is sufficient to validate the reliability of results [64–66]. However, the remaining 2.34% of nonmacrophage cells, particularly neutrophils, may exert negligible influences on the experimental outcomes. To determine the extent of the impact on the related results by nonmacrophage cells, further in-depth research is needed.

To further test whether this mesh was a form of MET, we used PicoGreen, a highly sensitive fluorescent nucleic acid dye, to accurately quantify dsDNA in the supernatants. An examination of the dsDNA produced by macrophages stimulated with *P. hominis* at different doses and for different durations revealed that the dsDNA content was significantly greater in the coincubation group than in the control group in a time-dependent manner. However, when several parasites stimulate immune cells, necrosis of the cells occurs, with cell membrane lysis resulting in the release of intracellular LDH and nucleic acids from the cytoplasm [67, 68], which affects the judgment of the type of macrophage death, as well as the detection of dsDNA released during the formation of METs. To exclude these effects, we examined the LDH content in the supernatant after coincubation of *P. hominis* with macrophages and found that the macrophages did not develop significant necrosis. Thus, *P. hominis* induces the release of METs from macrophages.

To further clarify the structure and composition of *P. hominis*-induced METs, we examined the structure of classical METs consisting of H3- and MPO-decorated DNA backbones via fluorescence microscopy [24, 58, 69]. The current study revealed that ETs from different sources share common features, including DNA backbones containing embedded antimicrobial peptides, proteases, and histones [24, 69]. MPO is an important iron-containing lysosome found in the peripheral blood, small intestine, large intestine, mesenteric lymph nodes, and undifferentiated macrophages and is an important component of the innate immune system [70, 71]. Furthermore, MPO kills phagocytic pathogens inside the cell and can be released outside the cell to destroy various target substances. MPO is also an important component of METs [72, 73], and all parasite-induced METs involve MPO [34–36]. In the present study, fluorescence confocal microscopic analysis and DNase I treatment confirmed that the main components of these reticulations were the DNA backbone, as well as H3 and MPO, which were specifically recognized and bound by H3 and MPO antibodies and colocalized extracellularly with Hoechst 33342-stained DNA. The macrophages were further pretreated with the MPO inhibitor ABAH and showed a significant decrease in dsDNA release after stimulation with *P. hominis* trophozoites, suggesting that MPO is required for MET formation. Thus, the DNA backbone of *P. hominis*-induced METs is modified by H3 and MPO, similar to the major components of METs induced by other lumen-parasitic protozoa [35, 36].

Recent study has revealed that macrophages release METs through a multistep METosis process that can be categorized into two distinct mechanisms: “NADPH-dependent” and “non-NADPH-dependent” [15]. NADPH oxidase is the key enzyme involved in “NADPH-dependent” processes [18]. When macrophages encounter pathogens or other activating stimuli, activated NADPH oxidase stimulates the production of ROS in macrophages, and increased ROS levels in macrophages trigger DNA depolymerization and chromatin release from the nucleus [74]. Moreover, METs can also be formed in a ROS-independent manner [33, 75]. Therefore, detecting the amount of ROS released and the role of NADPH oxidase during MET formation is key to determining the formation mechanism. Herein, we examined the release of ROS during the stimulation of MET formation by *P. hominis*. *P. hominis* stimulation of macrophages resulted in significantly higher levels of ROS in the cell supernatant than those in the control group, and the formation of *P. hominis*-induced METs was significantly reduced when NADPH oxidase activity was inhibited by the NADPH inhibitor DPI. These findings suggest that ROS are released in large quantities when *P. hominis* stimulates macrophages and are induced in METs via an NADPH-dependent pathway, similar to the results reported for several protozoan parasites that stimulate the formation of METs via the NADPH pathway [35, 36].

METosis is a complex process, and current studies have not fully elucidated the mechanism of parasite-induced MET, which may involve a variety of factors in addition to NADPH oxidase, which is required for MET regulation. PAD4 reportedly plays an important role in the formation of ETs, and the involvement of PAD4 has been reported in studies on METs induced by bacteria, fungi, and other stimuli but has not yet been reported in parasite-induced METs. PAD4-dependent citrullination of histone H3 is a component of ETs and is involved in a key molecular event during the formation of ETs [58]. PAD4 has a typical nuclear localization signal (NLS) that translocates into the nucleus and citrullinates histone H3, leading to chromatin depolymerization and release into the extracellular space to form ETs [47]. In this study, a significant reduction in *P. hominis*-induced METs was observed after the pretreatment of macrophages with the PAD4 inhibitor Gsk484, suggesting that PAD4 is involved in this process. This result is similar to most findings with respect to the mechanisms of MET formation [48, 50, 76], and this is the first time that PAD4 has been found to be involved in the resistance of METs to parasitic infection.

Numerous studies have shown that ET formation is dependent on SOCE [57] and that elevated intracellular calcium levels induced a Ca^2+^-dependent increase in PAD4 activity, which leads to histone citrullination. Moreover, ROS production is dependent on SOCE [58–60, 62]. The formation of parasite-induced METs is also dependent on SOCE. Herein, we used the SOCE inhibitor 2-APB to inhibit SOCE, which resulted in a significant reduction in MET formation. This finding suggest that SOCE plays a key role in *P. hominis*-induced MET formation in mice.

In recent years, several TLRs have been shown to be involved in NETosis [43, 77, 78], especially TLR2 and TLR4, which have been shown to be involved in the parasite-induced formation of NETs [44, 56]. Thus, TLRs and their associated signaling pathways play crucial roles in host cell resistance to parasitic infections. However, the role of TLRs in parasite-induced MET formation remains unclear. Therefore, the present study examined the expression of *Tlr2* in mouse peritoneal macrophages stimulated with different durations of the ET-positive stimulant zymosan. The results revealed that the expression of *Tlr2* significantly increased after macrophages were stimulated with zymosan, indicating that TLR2 is involved in the regulation of macrophage MET formation. The level of *Tlr2* gene expression significantly increased in macrophages after *P. hominis* stimulation, suggesting that TLR2 is also involved in *P. hominis*-induced MET formation. Further treatment of *P. hominis*-stimulated macrophages with the TLR2 inhibitor (C29) resulted in a significant decrease in the amount of dsDNA, which further demonstrated the involvement of TLR2 in the generation of *P. hominis*-induced METs. This is the first study to demonstrate the involvement of TLR2 in parasite-induced METs.

The Raf/ERK pathway is involved in the formation of NETs induced by phorbol 12-myristate 13-acetate (PMA) [79]. As a signaling pathway downstream of TLR2, the MAPK pathway is among the most conserved signaling pathways in eukaryotes [80]. To date, the three main MAPK families are ERK1/2, p38 MAPK, and stress-activated protein kinase (SAPK/JNK) [81]. SOCE is tightly regulated by ERK1/2 MAPK phosphorylation, and the ROS-dependent activation of ERK and p38 MAPK mediates the PMA-induced release of human neutrophil NETs [79]. In the present study, pretreatment of macrophages with ERK1/2 and p38 inhibitors (U0126 and SB202190) significantly inhibited the formation of *P. hominis*-induced METs, suggesting the importance of ERK1/2 and p38 MAPK in the formation of *P. hominis*-induced METs, which is similar to the results of related protozoan-induced METs.

During infection, ETs play an important role in assisting immune cells in capturing and killing pathogens, and it has been shown that macrophages can immobilize and kill parasites by releasing METs. However, one of the main functions of macrophages as immune cells is phagocytosis. To exclude the effect of phagocytosis on the capture of *P. hominis* by METs, we used Cyt D to inhibit phagocytosis by macrophages. To investigate the killing effect of METs on *P. hominis*, we examined the morphology and survival of *P. hominis* after coincubation with macrophages. Compared with that of normal *P. hominis*, the integrity of the cell membrane of *P. hominis* captured by METs was disrupted, the cell membrane was wrinkled, and many pores appeared. The survival rate of *P. hominis*, which was determined via trypan blue staining, revealed that the formation of METs decreased the number of surviving *P. hominis*. The lowest survival rate of *P. hominis* in this test was 69.28%, and the killing effect was in both time- and dose-dependent manner. The results of DNA release revealed that the release of METs was not related to the dose of *P. hominis*; however, the killing effect of METs on *P. hominis* was dose-dependent. This may be because MET formation has a threshold effect, which means macrophages trigger ETosis when parasites reach a certain number or stimulus intensity, and further increasing the parasite proportion does not linearly increase MET release. However, the killing efficiency of METs may be related to the density of pathogens. Although the total number of METs is fixed, an increase in the number or density of parasites increases the probability of capture, and a single release of METs can capture multiple parasites at the same time. This finding is similar to reports that bladder-resident macrophages release extracellular traps (METs) to block urinary tract pathogens, where the efficiency of pathogen clearance by METs is positively correlated with the bacterial or parasite load [82]. Thus, METs have a killing effect on *P. hominis*, suggesting that host METs play a role in resisting *P. hominis* infection.

## Conclusions

The results of this study revealed that *P. hominis* activates TLR2 to regulate the ERK1/2 and p38 MAPK signaling pathways and generates ROS via PAD4 and SOCE. TLR2 also regulates the activation of NADPH oxidase, which causes MPO and H3 translocation and the release of DNA to form METs capable of trapping and killing *P. hominis*. This is the first study to reveal that PAD4 and TLR2 are involved in parasite-induced METs. These findings provide important clues for research on the role of host innate immunity against *P. hominis* infections, thus laying the foundation for an in-depth study of the mechanism of host innate immunity against parasitic infections.

## Data Availability

All the data generated in the paper are available in the paper itself.
